# Role of the very low frequencies of the renal oxygen saturation signal in acute kidney injury in newborns with perinatal asphyxia

**DOI:** 10.3389/fped.2025.1490321

**Published:** 2025-01-20

**Authors:** Daniel Botero-Rosas, Sergio Agudelo-Pérez, Gloria Troncoso, Maria C. Gómez, Eduardo Tuta-Quintero

**Affiliations:** ^1^ School of Medicine, Universidad de La Sabana, Chía, Colombia; ^2^ Neonatal Intensive Care Unit, Fundación Cardio Infantil—Instituto de Cardiología, Bogotá, Colombia

**Keywords:** renal autoregulation, very low-frequency oscillations, acute kidney injury, neonatal asphyxia, infant, newborn

## Abstract

**Objective:**

Renal autoregulation, hemodynamic response, and endothelial dysfunction play significant roles in acute kidney injury (AKI) during perinatal asphyxia. A third mechanism of autoregulation, involving very low-frequency oscillations, has been described. This study aimed to evaluate the relationship between the power of the very low-frequency component of the Fast Fourier Transform (FFT) and AKI during therapeutic hypothermia (TH) treatment in neonates with perinatal asphyxia.

**Study design:**

A retrospective longitudinal study was conducted on neonates with moderate and severe perinatal asphyxia. AKI was defined as a decrease of less than 33% in the serum creatinine level by day 3. The power of the very low-frequency component in the FFT was assessed by analyzing renal oxygen saturation using near-infrared spectroscopy (NIRS), focusing on a frequency band of approximately 0.01 Hz. Bivariate analyses were performed to explore the association between the power of the very-low-frequency component and AKI. The predictive ability of this component for AKI was evaluated using a receiver operating characteristic (ROC) curve. Additionally, a generalized estimating equation (GEE) was developed to investigate whether changes in the power of the very-low-frequency component during treatment differed according to the presence of AKI.

**Results:**

A total of 91 patients were included in the study, of whom 15 (16.5%) developed AKI. Neonates with AKI exhibited a significantly lower power of the very low-frequency component on the second day of treatment (*p* = 0.001). This component demonstrated good predictive ability for AKI (ROC curve 0.77, 95% CI 0.63–0.90).

**Conclusion:**

Among neonates with perinatal asphyxia who developed AKI, a lower power of the very-low-frequency component in FFT (approximately 0.01 Hz) was observed on the second day of therapeutic hypothermia. This finding suggests that alterations in very-low-frequency oscillations may reflect endothelial dysfunction and contribute to the development of AKI, warranting further investigation in larger cohorts.

## Introduction

Perinatal asphyxia (PA) results from impaired blood flow and gas exchange due to an acute event occurring near birth (1). With an overall incidence of 4.4 per 1,000 live births, it is the second leading cause of death in the immediate neonatal period (2). Furthermore, it is the primary cause of both short- and long-term neurological consequences in full-term neonates (3). In addition, other organs are frequently affected by asphyxia, which increases the risk of mortality, with the kidneys being one of the most affected organs (4, 5). Acute kidney injury (AKI) occurs in 50%–72% of neonates with perinatal asphyxia (4). Additionally, the risk of long-term renal function complications increases in surviving neonates (6). Although treatment with therapeutic hypothermia reduces the incidence of AKI, it remains a strong predictive factor in neonates with asphyxia (7).

Hemodynamic responses secondary to asphyxia include the redistribution of blood flow to vital organs and decreased systemic and renal blood flow (8, 9). Severe and prolonged renal hypoperfusion resulting from asphyxia can lead to acute renal injury, acute tubular necrosis, and endothelial cell dysfunction (10). Previous studies using renal Doppler have reported a direct relationship between increased renal artery resistance and decreased renal blood flow (RBF) with the development of AKI in children with perinatal asphyxia (11).

Similarly, it has been shown that renal autoregulatory mechanisms are altered during asphyxia, leading to renal hemodynamic changes associated with AKI (12). Autoregulation of RBF is controlled by both extrinsic and intrinsic mechanisms. Intrinsic mechanisms are mediated by two classical components: myogenic regulation and tubuloglomerular feedback (TGF) (13, 14). Furthermore, animal studies and mathematical modeling have proposed a third intrinsic endothelial mechanism of slow regulation that oscillates at very low frequencies (approximately 0.01 Hz) and interacts with the other two to regulate RBF (15, 16).

Recent evidence suggests that even lower-frequency oscillations (∼0.01 Hz), mediated by endothelial mechanisms, may play a critical role in regulating vascular tone and ensuring consistent perfusion (17, 18). These lower-frequency oscillations are sensitive to pathophysiological changes such as congestive heart failure and endothelial dysfunction, suggesting their potential as early markers of vascular abnormalities (18). Analysis of these oscillations using advanced techniques, such as wavelet transforms and laser Doppler flowmetry (LDF), has revealed distinct patterns that differentiate endothelial contributions from other hemodynamic components (18, 19). Specifically, the amplitude of these oscillations increases in response to endothelium-dependent stimuli, such as acetylcholine administration, confirming their origin in endothelial activity mediated by nitric oxide (NO) (19). In pathological conditions such as heart failure, the reduction of these oscillations and their partial restoration with β1-blockers highlight their sensitivity to endothelial function and their potential as non-invasive biomarkers (18). These findings suggest that very low-frequency oscillations could represent an additional layer of vascular autoregulation with significant clinical implications (17).

The development of AKI has traditionally been described in four phases. Initially, a decrease in RBF leads to tubular epithelial necrosis (initiation phase). Endothelial dysfunction and injury were observed during the subsequent extension phase. Depending on the severity and extent of the injury, the maintenance and recovery phases follow (17). Endothelial injury and dysfunction described in the extension phase may be associated with the involvement of a third autoregulatory mechanism, and could contribute to AKI in neonates with perinatal asphyxia. Moreover, changes in the energy at very low frequencies (∼0.01 Hz) may reflect dynamic alterations in the power of the third autoregulatory mechanism related to the endothelium (16, 18, 19). This phenomenon may be linked to the pathophysiology of AKI.

Therefore, we hypothesized that a decrease in the power of the very-low-frequency component of the NIRS signal is associated with the development of AKI in neonates with asphyxia treated with therapeutic hypothermia. This study aimed to determine the relationship between very-low-frequency oscillations in NIRS signals and the development of AKI during therapeutic hypothermia in neonates with perinatal asphyxia. Recognizing changes in the power of the very low-frequency component of the NIRS signal could help elucidate the role of the third intrinsic mechanism in the pathophysiology of neonatal AKI.

## Methods

The data for the analysis presented in this study were obtained from an original study conducted to evaluate the relationship between renal oxygen saturation (rSrO2), measured using near-infrared spectroscopy (NIRS), and AKI in neonates with perinatal asphyxia. The study protocol was outlined as follows:

A retrospective longitudinal study was conducted in neonates with perinatal asphyxia and indications for therapeutic hypothermia (TH) admitted to the neonatal intensive care unit (ICU) at Fundación Cardioinfantil, Instituto de Cardiología, between November 2021 and November 2022. The study protocol was approved by the institution's ethics and research committee and registered under number CEIC-0602-2022. Because this was a retrospective study, informed consent was not obtained from the participants. In accordance with national regulations and ethics committee guidelines, data collection and analysis were conducted to ensure confidentiality and protection of patient information.

Full-term and near-term neonates (Ballard ≥36 weeks) with postnatal age ≤12 h, moderate and severe perinatal asphyxia, and indications for therapeutic hypothermia according to the criteria of the Colombian Association of Neonatology (ASCON, as per its acronym in Spanish) were included (20). The diagnosis of severe perinatal asphyxia was confirmed by the presence of at least three of the following criteria: an Apgar score <5 at 5 min, umbilical cord blood gases or peripheral blood collection within the first hour of life with pH <7.0, base deficit ≤−16 mmol/L, lactate ≥12 mmol/L, and moderate or severe encephalopathy according to Sarnat stages II or III. Moderate asphyxia was confirmed by at least two of the following criteria: mild or moderate encephalopathy according to Sarnat stages I or II, an Apgar score ≤7 at 5 min, and umbilical cord blood gasometry or peripheral blood draw in the first hour of life with a pH ≤7.15. Newborns with major congenital or genetic anomalies incompatible with life, severe intrauterine growth retardation with a birth weight <1800g, or congenital renal or urinary anomalies were excluded from the study.

The hypothermia protocol was performed according to recommended guidelines (21). The main outcome of this study was the presence of AKI 72 h after the initiation of TH. AKI was defined based on recommendations for patients with perinatal asphyxia who were treated with TH (22). AKI was defined as a decrease in serum creatinine level of <33% between admission to the unit and the third day (72 h). Clinical data were obtained from patients’ electronic health records for characterization and analysis.

### NIRS signal acquisition and processing

The data were processed and analyzed using the following steps:

• Data Acquisition: rSrO2 monitoring was performed using the NIRS-INVOSTM neonatal system with an oximetry sensor placed below the costal margin and above the iliac crest, with the sensor tip lateral to the spine, and the reading end of the sensor wrapped around the body. The device recorded rSrO2 every 30 s.
• Data Storage: The data were stored on the device and exported to an Excel database using the INVOS software, Shortcut to Invos Analytics Tool®. Missing data from the renal rSrO2 time series (TS) were identified and estimated using linear interpolation. The matrices were then homogenized in duration with the last value of the time series, ensuring the analysis of TSs with an equal amount of data for all neonates. The rSrO2 TSs were subsequently loaded into MATLAB R2023b for further analyses.
• Extraction of Parameters and Analysis in the Frequency Domain: Initially, the linear trend of the rSrO2 time series for neonates, with and without AKI, during 24 h periods, was removed from all signals. Subsequently, the power spectral density (PSD) of the rSrO2 time series was calculated in the frequency band related to endothelial activity (very low-frequency band: 0.007–0.02 Hz) using the Welch periodogram method. The following specifications were considered in this calculation:
  ○ Window length: 500 samples
  ○ Overlap: 300 samples or 60%
  ○ FFT length: 500 points, as defined by the Welch periodogram method
  ○ Sampling frequency: 0.033 Hz, which corresponds to the sampling frequency of the NIRS device for rSrO2.
• The energy in the Very Low Frequency (VLF) band was then obtained through trapezoidal integration of the Power Spectral Density (PSD) function.

### Statistical analysis

Qualitative variables were described using absolute and relative frequencies. Quantitative variables were described using measures of central tendency and dispersion depending on their distribution. The distribution assumption was evaluated using the Shapiro-Wilk test.

The independent variable was the power of the VLF component, and the dependent variable was the presence of AKI 72 h after TH treatment. The association between the power of the VLF and AKI was assessed daily using the Mann-Whitney U test. To assess the predictive capacity of VLF power for the presence of AKI on the third day, receiver operating characteristic (ROC) curves were constructed for each day, analyzing the area under the curve (AUC).

To explore whether the change in VLF power over time during therapeutic hypothermia differed between patients with and without AKI, a generalized estimating equation (GEE) was used. Statistical significance was considered for *p*-values ≤ 0.05 for all analyses. Analyses were performed using MATLAB and IBM SPSS 21.

## Results

**Figure 1:**
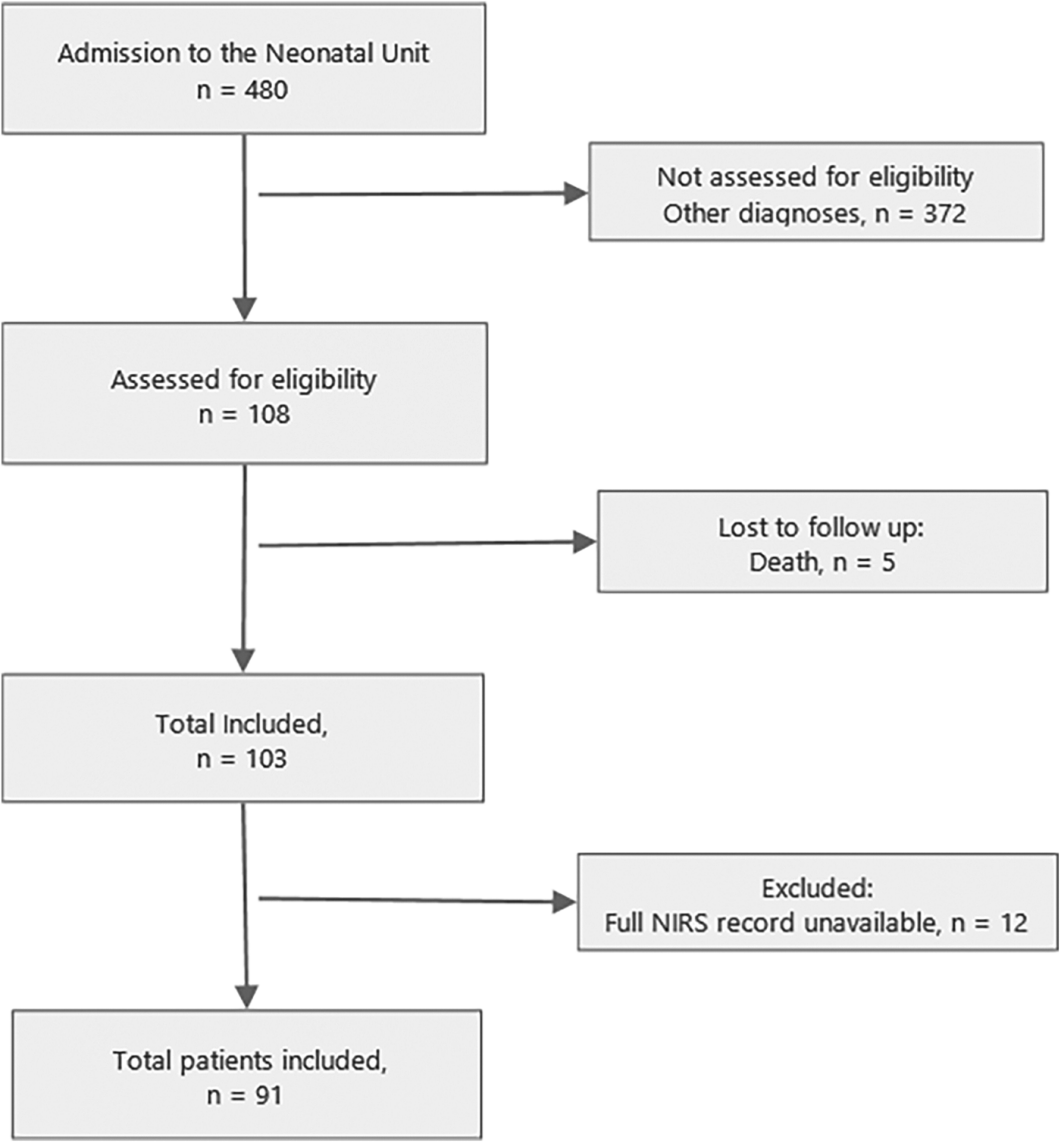
Flow diagram of study. Note: NIRS, near-infrared spectroscopy.

A total of *n* = 91 patients were included (Figure 1). Of these, 15 (16.5%) developed AKI. Differences were found between groups according to the presence of AKI in Apgar score at 1 min (*p* = 0.02) and 5 min (*p* = 0.03), use of inotropes (*p* = 0.05), pH value (*p* = 0.04), HCO3 (*p* = 0.03), and base excess (*p* = 0.05) in cord blood gases (Table 1).

**Table 1:** Clinical characteristics of patients.

Variable	Acute kidney injury				*P*-value
	No (*n* = 76)		Yes (*n* = 15)		
Sex, *n* (%)					
Female	31	(79.5)	8	(20.5)	0.37
Male	45	(86.5)	7	(13.5)	
Gestational age—Ballard, *n* (%)					
≥37 Weeks	66	(82.5)	14	(17.5)	0.68^a^
<37 Weeks	10	(90.9)	1	(9.1)	
Birth weight—Grams, *n* (%)					
≥2,500	63	(82.9)	13	(17.1)	1.0^a^
<2,500	13	(86.7)	2	(13.3)	
Birth, *n* (%)					
Vaginal	40	(85.1)	7	(14.9)	0.67
Cesarean section	36	(81.8)	8	(18.2)	
Resuscitation at birth, *n* (%)					
No	35	(87.5)	5	(12.5)	0.32
Yes	39	(79.6)	10	(20.4)	
APGAR score at first minute, *n* (%)					
>5	39	(92.9)	3	(7.1)	0.02
≤5	37	(75.5)	12	(24.5)	
APGAR score at fifth minute, *n* (%)					
>5	71	(86.6)	11	(13.4)	0.03^a^
≤5	5	(55.6)	4	(44.4)	
Asphyxia, *n* (%)					
Moderate	29	(87.9)	4	(12.1)	0.39
Severe	47	(81.0)	11	(19.0)	
Encephalopathy—sarnat, *n* (%)					
Moderate	67	(85.9)	11	(14.1)	0.21^a^
Severe	9	(69.2)	4	(30.8)	
Use of inotropic agents, *n* (%)					
No	36	(92.3)	3	(7.7)	0.05
Yes	40	(76.9)	12	(23.1)	
Cord blood gases, mean (sd)					
pH	6.97	(0.11)	6.90	(0.1)	0.04
HCO3	12.27	(4.7)	9.2	(5.7)	0.03
Base excess	−17.3	(4.9)	−20.1	(5.8)	0.05
Lactate	10.9	(4.1)	12.9	(4.6)	0.08
Hemoglobin (mg/dl), mean (ds)	17.6	(2.3)	17.7	(1.9)	0.93

Notes: m, median; SD, standard deviation; IQR, interquartile range.

^a^ Fisher's Exact Test.

**Figure 2:**
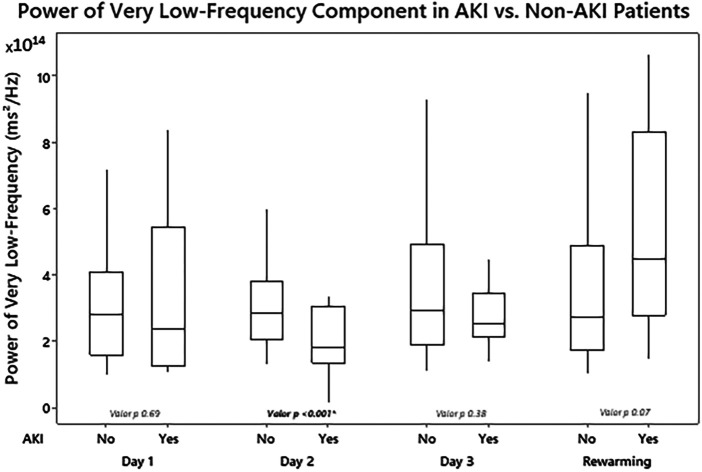
Differences in renal power of the very low-frequency component of fast Fourier transformation (FFT) by measurement periods according to the presence or absence of acute kidney injury.

**Figure 3:**
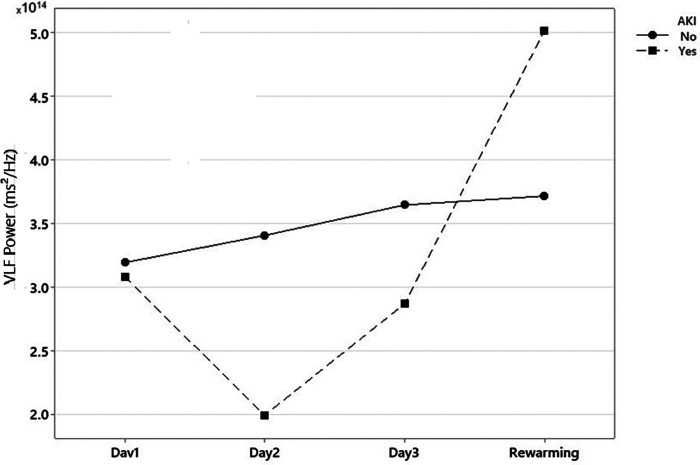
Global changes in power of the very low-frequency component of fast Fourier transformation (FFT) during therapeutic hypothermia. Note: AKI, acute kidney injury. *Power of the very low-frequency component of Fast Fourier Transformation (FFT) in neonates with and without Acute Kidney Injury during treatment with Therapeutic Hypothermia.

Significant statistical differences were found in the power of the VLF component between patients with and without AKI on day 2 of TH (median—RIQ): 2.87 × 10^14^ (1.73 × 10^14^) vs. 1.65 × 10^14^ (1.55 × 10^14^), *p* = 0.001 (Figure 2). Additionally, the power of the VLF component on day 2 had a strong ability to predict the presence of AKI on day 3 (AUC 0.77, 95% 0.63–0.90) (Table 2). In the GEE model, no significant differences were observed in the changes in the power of the VLF during the three days of treatment between the two groups of patients (coefficient 4.87 × 10^13^; IC 95% −3.1 × 10^12^, 1 × 10^14^, *p*-value 0.06) (Figure 3; Table 3).

**Table 2:** Receiver operating characteristic curve at each time during hypothermia treatment.

Power of the very low-frequency component of fast fourier transformation	ROC-Curve	CI 95%		*p*-value
Day 1	0.58	0.41	0.76	0.33
Day 2	0.77	0.63	0.90	0.001
Day 3	0.56	0.44	0.68	0.46

Notes: ROC-Curve, receiver operating characteristic curve; CI, confidence interval.

**Table 3:** Generalized estimating equation analysis for power of the very low-frequency component of fast Fourier transformation evolution during therapeutic hypothermia.

Power of the very low-frequency component of fast fourier transformation	Coefficient	CI 95%		*p*-value
Acute kidney injury				
Yes	4.87 × 10^14^	−3.1 × 10^13^	1 × 10^14^	0.06
Constant	3.04 × 10^14^	2.4 × 10^14^	3.62 × 10^14^	

Note: CI, confidence interval.

## Discussion

This study evaluated the association between very-low-frequency oscillations (power of the VLF, approximately 0.01 Hz) and AKI in neonates with asphyxia treated with TH. The results showed that Neonates who developed AKI exhibited significantly lower VLF power on day 2 of TH. These findings not only confirm the presence of endothelial dysfunction during the extension phase of AKI, but also provide direct evidence of altered renal autoregulatory mechanisms in human neonates with asphyxia. This offers a unique perspective that bridges experimental findings from animal models with clinical observations in neonates. This study represents a significant advancement as it is the first to explore this association in human neonates through VLF band energy analysis of the rSrO2 signal obtained with NIRS, suggesting the involvement of a potential third renal autoregulatory mechanism dependent on the endothelium in the pathogenesis of AKI.

In the context of renal autoregulation, the interaction between the myogenic mechanism (fast) and tubuloglomerular feedback (TGF, slow) has traditionally been described as the primary mechanism for maintaining renal blood flow (RBF) homeostasis (23, 24). However, studies in animal models have identified a third mechanism, characterized by oscillatory behavior at very low-frequency oscillations (∼0.01 Hz), which could play a modulatory role in renal autoregulation (16, 25). This mechanism, first described by Siu et al., appears to interact with myogenic and TGF mechanisms, complementing their functions under both normal and pathological conditions (15, 16). Similarly, both Siu (15) and Just (16) suggested the existence of a third autoregulatory mechanism, likely endothelial in nature, operating at very low frequencies (∼0.01 Hz) and potentially interacting with myogenic (0.1–3 Hz) and TGF (0.02–0.5 Hz) mechanisms. Our findings demonstrate that these VLF oscillations are significantly diminished in neonates with AKI, suggesting that endothelial dysfunction may impair the modulatory capacity of this third mechanism and exacerbate renal hemodynamic instability. Although the current evidence does not establish direct modulation, both studies propose that this third mechanism contributes to the autoregulatory dynamics of renal blood flow, complementing the rapid responses of the other two mechanisms. Furthermore, previous studies have suggested that this mechanism depends on endothelial processes, such as nitric oxide (NO) release and angiotensin II modulation, highlighting its sensitivity to hemodynamic alterations and its potential impact on the pathophysiology of AKI (16, 26).

Our results indicate that alterations in the VLF band of the rSrO2 signal obtained with NIRS in neonates with AKI may reflect endothelial dysfunction during the extension phase of AKI, which is characterized by the damage and dysfunction of endothelial cells (15, 26). This is consistent with findings in patients with chronic kidney disease (CKD), where oscillations around ∼0.0095 Hz of renal blood flow were significantly altered, even in the absence of concomitant cardiovascular disease or diabetes (26). The reduction in VLF power observed on day 2 of TH in neonates with AKI provides a plausible explanation for how endothelial dysfunction disrupts autoregulatory balance, potentially influencing renal perfusion and contributing to AKI progression. These results highlight a critical aspect of renal pathophysiology in asphyxiated neonates that has not been addressed previously. Hypoperfusion and cardiovascular dysfunction are observed in neonates with severe asphyxia, and endothelial damage can disrupt this third autoregulatory mechanism, contributing to renal injury progression and its association with AKI.

This third autoregulatory mechanism is postulated to depend on pre-glomerular renal endothelial control, which modulates the influence of nitric oxide and angiotensin II (27). Moreover, during the extension phase of acute kidney injury (AKI), which manifests after 24 h, endothelial cell dysfunction and death are observed (28). This finding suggests that endothelial injury plays a key role in the development of ischemic AKI (28–30). A significant percentage of neonates in the cohort studied presented with severe asphyxia and cardiovascular dysfunction, with a high need for vasopressor support, explaining hypoperfusion and renal hypoxia from a hemodynamic standpoint. This situation likely explains the renal endothelial damage observed in this study through the analysis of the VLF power in the rSrO2 signal obtained with renal NIRS. Furthermore, given that this third mechanism seems to have endothelial dependence in its control, the endothelial dysfunction observed in asphyxia may alter this mechanism, leading to hemodynamic and renal perfusion alterations and the observed association with AKI.

An additional finding of this study was that the power of the VLF component of the rSrO2 signal obtained with renal NIRS during the rewarming phase was significantly higher in the AKI group than in the non-AKI group. This result could be interpreted as a compensatory effort by the endothelium in response to more severe damage, where vasodilation induced by nitric oxide (NO)-dependent mechanisms attempts to counteract renal hypoperfusion. However, this increased energy could also reflect dysfunctional hyperactivity that fails to adequately restore renal perfusion, necessitating further investigation to clarify this phenomenon.

Additionally, the predictive capacity of the VLF on day 2 (AUC 0.77) suggests that these oscillations could serve as noninvasive biomarkers to identify neonates at risk of developing AKI. This finding aligns with those of previous studies that identified VLF in renal blood flow as a sensitive marker of vascular dysfunction in various renal conditions (15, 26, 31).

Thus, an endothelial role in controlling renal intrinsic autoregulatory mechanisms has been proposed, with endothelial dysfunction during the extension phase likely associated with the involvement of a third autoregulatory mechanism. This contributes to renal hypoperfusion, the extension of injury, and progression to AKI. Our study provides critical clinical evidence supporting this hypothesis and emphasizes the need for future research to further explore the precise contribution of these mechanisms to the renal pathophysiology in neonates with asphyxia. However, no previous human studies have explored this renal autoregulatory mechanism and we believe that future studies should elucidate its role in controlling renal blood flow and its involvement in AKI development under various conditions.

The limitations of this study include the fact that the original design evaluated rSrO2 levels in AKI in neonates with asphyxia. However, the analysis of the power of the VLF component was based on the rSrO2 recordings, which was the original study design. Additionally, the NIRS sampling frequency (every 30 s) allowed for the analysis of only very-low-frequency oscillations (∼0.01 Hz). A broader view encompassing other flow control systems would require a higher sampling rate of the device, with respect to the Nyquist frequency, to observe the physiological frequency components of the renal blood flow control system.

## Conclusion

A lower power of the VLF component of the rSrO2 signal obtained with renal NIRS was observed in neonates with asphyxia who developed AKI on the second day of treatment with therapeutic hypothermia. This finding supports the hypothesis that endothelial dysfunction may be associated with AKI development by potentially disrupting this third autoregulatory mechanism, as suggested by experimental studies in animal models. However, the lack of significant differences in the longitudinal changes in these oscillations across the three days of treatment highlights the complexity of their dynamics and suggests the need for cautious interpretation. These results add to the understanding of endothelial dynamics but require further validation in studies designed to explore the full spectrum of renal autoregulatory mechanisms.

Future research should consider prospective longitudinal designs with higher rSrO2 sampling frequencies to capture the complete frequency band in which different mechanisms of renal blood flow regulation operate, enabling a more comprehensive characterization of their interactions and contributions to the AKI pathophysiology.

## Data Availability

The raw data supporting the conclusions of this article will be made available by the authors, without undue reservation.
